# A systematic review and meta-analysis of the diagnostic accuracy of self-report screening instruments for common mental disorders in Arabic-speaking adults

**DOI:** 10.1192/j.eurpsy.2022.276

**Published:** 2022-09-01

**Authors:** A. De Graaff, P. Cuijpers, M. Leeflang, I. Sferra, J. Uppendahl, M. Sijbrandij, R. De Vries

**Affiliations:** 1 Vrije Universiteit Amsterdam, Clinical, Neuro- And Developmental Psychology, Amsterdam, Netherlands; 2 University of Amsterdam, Department Of Epidemiology And Data Science, Amsterdam, Netherlands; 3 Sapienza University of Rome, Department Of Human Neurosciences, Rome, Italy; 4 Vrije Universiteit Amsterdam, Medical Library, Amsterdam, Netherlands

**Keywords:** diagnostic test accuracy, questionnaires, Common mental disorders, sensitivity/specificity

## Abstract

**Introduction:**

Self-report questionnaires to screen for symptoms of common mental disorders (CMDs) are commonly used as inexpensive, easy-to-administer tools in research and clinical practice. However, their validity to detect the presence of any CMD across cultures and languages is unclear. Psychometrically sound and brief case-finding instruments are vital for the identification of individuals with mental health needs. With the increasing number of Arabic-speaking refugees in Europe, we aim to evaluate the diagnostic accuracy of Arabic-language screening instruments.

**Objectives:**

The aim of this systematic review/meta-analysis is to synthesize the diagnostic accuracy of self-report questionnaires to detect depression, anxiety and posttraumatic stress disorder (PTSD) in Arabic-speaking populations.

**Methods:**

Five databases were searched (inception-January 2021) (PROSPERO: CRD42018070645) for studies on the diagnostic accuracy of brief questionnaires in Arabic-speaking populations, with a clinical interview as reference standard. Data on sensitivity/specificity were extracted/calculated. Multi-threshold meta-analyses were performed (R diagmeta package). Study quality was assessed using QUADAS-2.

**Results:**

We included 32 studies (N=4042 participants) reporting on questionnaires targeting depression/anxiety (14 questionnaires), distress (2 questionnaires), and PTSD (1 questionnaire). Optimal thresholds were identified for the Edinburgh Postnatal Depression Scale (EPDS; cut-off 11, sensitivity 76.9%, specificity 85.1%), Hospital Anxiety and Depression Scale (HADS) anxiety subscale (cut-off 7, sensitivity 81.9%, specificity 87.6%), depression subscale (cut-off 6, sensitivity 73.0%, specificity 88.6%), and Self-Reporting Questionnaire (SRQ-20; cut-off 8, sensitivity 86.0%, specificity 83.9%).

**Conclusions:**

We present optimal thresholds that can be used by clinicians and researchers for the EPDS, HADS and SRQ-20. More research on Arabic-language questionnaires, especially those targeting PTSD, is needed.

**Disclosure:**

No significant relationships.

